# Co-expression gene module analysis in response to attenuated cercaria vaccine reveals a critical role for NK cells in protection against *Schistosoma mansoni*

**DOI:** 10.1186/s13071-024-06505-0

**Published:** 2024-11-19

**Authors:** Almiro Pires da Silva Neto, Juliana Vitoriano-Souza, Mariana Ivo Khouri, Regiane Degan Favaro, Robert Alan Wilson, Luciana Cezar de Cerqueira Leite, Pablo Ivan Pereira Ramos, Leonardo Paiva Farias

**Affiliations:** 1 Laboratório de Medicina e Saúde Pública de Precisão (MeSP2), Instituto Gonçalo Moniz, Fundação Oswaldo Cruz, Salvador, Bahia Brazil; 2 Laboratório Especial de Desenvolvimento de Vacinas, Instituto Butantan, São Paulo, SP Brazil; 3 Departamento de Ciências da Vida, Universidade do Estado da Bahia, Salvador, Bahia Brazil; 4 Escola Bahiana de Medicina e Saúde Pública (EBMSP), Salvador, Bahia Brazil; 5 Department of Biology, York Biomedical Research Institute, University of York, York, UK; 6 Centro de Integração de Dados e Conhecimentos para Saúde (CIDACS), Instituto Gonçalo Moniz, Fundação Oswaldo Cruz, Salvador, Bahia Brazil

**Keywords:** Co-expression network, Radiation-attenuated vaccine, Hub-genes

## Abstract

**Background:**

Despite decades of research, an effective schistosomiasis vaccine remains elusive. The radiation-attenuated (RA) cercarial vaccine remains the best model for eliciting high levels of protection. We have recently explored this model in mice to identify potentially protective pathways by examining gene expression patterns in peripheral blood mononuclear cells (PBMC).

**Methods:**

Herein, we reanalyzed the transcriptomic data from PBMC obtained from vaccinated and infected C57BL/6 mice in three timepoints (Days 7 and 17 after infection or vaccination and Day 7 post-challenge). In addition, we generated new data on PBMC collected 35 days after infection. Deconvolution analysis was performed to estimate immune cell composition by CIBERSORTx. Gene co-expression networks and over-representation analysis (ORA) were performed using the CEMiTool package. Protein-protein interaction networks were constructed using STRING, and the hub proteins for each module were identified using Cytoscape.

**Results:**

Co-expression network analysis identified a module (M2) associated with the infection process, grouping genes related to a Th2 immune response, and a second module (M6) associated with the vaccination process, displaying pathways related to a Th1 response, CD8 + T cells and NK cells. Within each module, five hub proteins were identified based on protein-protein interaction networks. The M2 infection module revealed Chil3, Il4, Cx3cr1, Emr1 and Ccl2 as hubs, while module M6, associated with vaccination, disclosed Prf1, Klrc1, IFN-γ, Ncr1 and Tbx21 as hub proteins.

**Conclusions:**

Our data point to the potentiald role of NK cells that may contribute to the RA vaccine response through the production of IFN-γ orchestrated by the T-bet transcription factor (Tbx21).

**Graphical Abstract:**

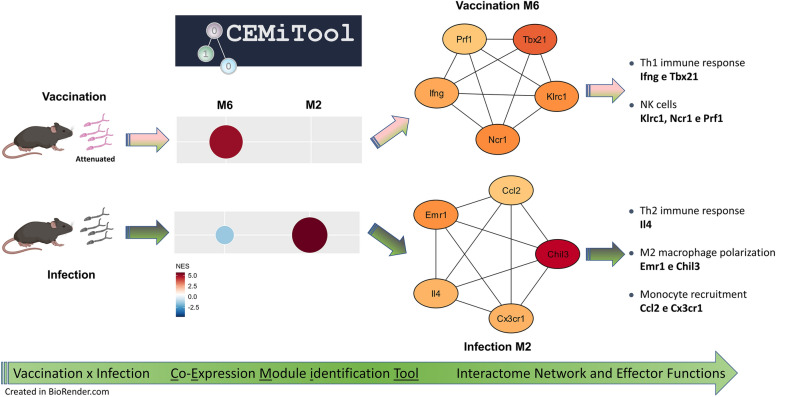

**Supplementary Information:**

The online version contains supplementary material available at 10.1186/s13071-024-06505-0.

## Background

Schistosomiasis is a neglected tropical disease caused by flatworms of the genus *Schistosoma*. Being mostly present in tropical and subtropical zones, schistosomiasis affects almost 240 million people with 779 million at risk [1]. Treatment with praziquantel (PZQ) is effective against the adult worm stage and has been used together with water supply, sanitation and hygiene (WASH) measures as the main strategies for disease control. Although the disease prevalence and worm burden have decreased in the last decades, schistosomiasis still represents a serious public health problem in persistent hotspots, especially because diagnostics for schistosomiasis cannot detect low-level infections [2]. In this context, an effective vaccine with long-term protective effects would be the ideal tool to prevent and ultimately eradicate this disease [3], a goal proposed in the WHA65.21 resolution endorsed by WHO member states in 2012 [4].

For many pathogens, the most effective vaccines comprise the live infectious agent that has been attenuated to make it non-pathogenic ([5, 6]). For schistosomes, studies with radiation-attenuated (RA) cercariae have demonstrated that a schistosomiasis vaccine is a realistic goal [reviewed in ([7])].

The RA vaccine model was first described by Minard et al. [reviewed in ([7])] and to date remains the gold standard for schistosomiasis vaccinology studies. The model brought a new perspective to vaccine development as it can confer up to ~ 75% effective protection with three vaccination doses [8]. The effector mechanism elicited by the RA cercarial vaccine relies on a pulmonary inflammatory response, involving cytokines such as IFN-γ and TNFα, to deflect challenge parasites into the alveoli (reviewed by Hewitson [3]). However, even after decades of the first report and testing of this vaccine in different animal models, no advances on how to mimic its immune responses using recombinant antigens have been made.

Systems vaccinology has unlocked a new pathway to study the molecular signatures related to immune events that occur after vaccination. Some studies have explored this approach for a series of marketed anti-viral or anti-bacterial vaccines against acute diseases (e.g. yellow fever, influenza and meningococcal diseases) and chronic diseases (e.g. malaria and HIV) (reviewed in [9]).

In the context of schistosomiasis vaccines, limited studies have examined the changes in host gene expression following vaccination and challenge assays. Tian et al. investigated the immunological events in peripheral blood mononuclear cells (PBMC) induced by vaccination of pigs with ultraviolet (UV)-attenuated cercariae of *S. japonicum*, revealing that IFN-γ, as well as genes related to cytotoxic Th1 response, are associated with higher protection levels [10]. Rojo et al. employed RNA sequencing to identify colony-stimulating factor (CSF), S100A alarmin and TNFRSF genes that may predict calpain vaccine efficacy in mouse and baboon models [11].

Recently, we employed a systems vaccinology approach to investigate the cercariae RA vaccine model [12]. We have assessed the transcriptional profiles of skin-draining lymph nodes (sdLNs) and PBMC at various timepoints following vaccination and challenge. Gene Set Enrichment Analysis (GSEA) revealed that vaccination activates pathways related to growth factors, whereas infection inhibits hemostasis pathways [12].

The GSEA approach tests for the enrichment of predefined gene sets constructed based on prior biological knowledge [13]. In contrast, gene co-expression networks identify groups of genes (i.e. modules) sharing similar expression patterns, resulting in a reduced gene universe that can be correlated with specified phenotypes [14] without relying on a priori knowledge.

Herein, we further explore the gene signatures triggered by vaccination and infection, employing gene co-expression network analysis. Additionally, we present new experimental data from PBMC collected at 35 days after a high-dose infection with 500 cercariae to better characterize the signatures associated with infection. Our findings revealed co-expression modules exclusively associated with infection or vaccination. Furthermore, we offer insights into potential gene hubs and effector mechanisms that merit exploration in the development of a vaccine against schistosomiasis.

## Methods

### Ethics statement

The study was carried out using C57BL/6 female mice aged 6–8 weeks, and all experiments were conducted in strict accordance with good practices as defined by the Committee for the Ethical Use of Animals in Experimentation of the Butantan Institute (São Paulo, Brazil) under license 1030/13.

### Experimental design

The current report is a companion study to the previous published systems biology analysis [12]. Herein, we have reanalyzed the transcriptome data provided by Farias (2021) (Gene Expression Omnibus accession number GSE164094) [12].

**Fig. 1:**
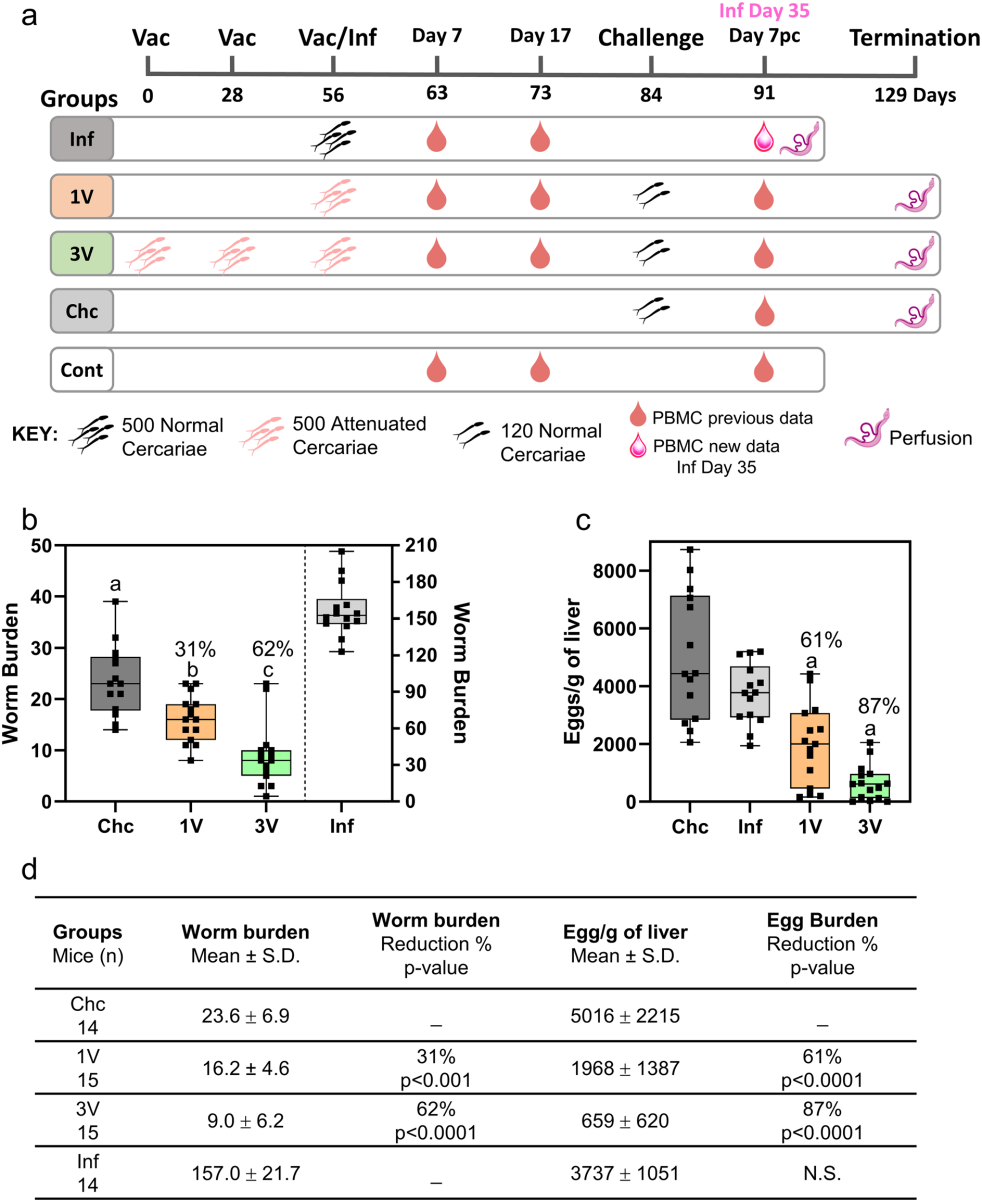
**a** The experimental design encompassing immunization, infection and termination procedures, along with specific timepoints for collecting PBMC for gene expression analysis. The Inf group at Day 35, incorporated in this study, is highlighted. **b** Box plot illustrating the worm burden on Day 129 for the Chc, 1 V and 3 V groups (each infected with 120 cercariae) and on Day 35 for Inf group (infected with 500 normal cercariae). **c** Box plot showing the egg burden for the same groups at the same timepoints as in **b**. Letters denote significant differences, and the percentage of reduction is indicated above the box plot. **d** Summary of parasitological data, including protection level, sample size and associated *p*-values, as determined by ANOVA and Tukey’s posttest (*p* < 0.05), for worm burden comparisons among Chc, 1 V and 3 V, and egg burden comparisons among Chc, 1 V 3 V and Inf

The data include the expression profile from peripheral blood mononuclear cells (PBMC) obtained from C57BL/6 female mice (*n* = 75), divided into five groups: the vaccinated once (1 V) group (*n* = 12), exposed to 500 attenuated cercariae and challenged with 120 normal cercariae; the vaccinated three times (3 V) group (*n* = 14), receiving 500 attenuated cercariae at 4-week intervals and challenged with 120 normal cercariae; the challenge-only (Chc) group (*n* = 12), subjected to 120 normal cercariae at 84 days; and the infected (Inf) group (*n* = 12), exposed to 500 normal cercariae concurrently with the 1 V group. The control (C) group of untreated mice (*n* = 22) was used to assess baseline gene expression. Samples were collected at three timepoints [Days 7 and 17 after infection or vaccination, and Day 7 post-challenge (Day 7 pc)], and a summary of the experimental design is presented in Fig. 1a. In addition, a new group/timepoint was incorporated into the analyses of the present study; this corresponds to PBMC transcriptomics data collected from mice 35 days after the high dose infection of 500 normal cercariae (Inf Day 35). A summary of the entire dataset used in this study, including worm burden and egg burden is provided in Additional file 1: Table 1. The reductions in worm and egg burden (%) in vaccinated mice were calculated by comparing the number of worms and number of eggs per gram of liver recovered in each vaccinated group with those in the Chc group, as previously described [12] and shown in Fig. 1b–d.

### Blood collection, PBMC separation, RNA extraction, amplification and labeling

Four independent biological replicates of PBMC from the infected group at Day 35 were processed for microarray analysis. All biological replicates consisted of a pool of PBMCs (containing equal amounts of total RNA) from three mice. Blood collection, PBMC separation, RNA extraction, quality control, amplification, labeling and hybridization were performed exactly as previously described [12] (Additional file 1: Table 1). The complete dataset containing 55 microarray samples was reanalyzed as described below.

### Microarray processing and calculation of differentially expressed genes

The raw microarray data were loaded using the read.maimage function from the limma package in R/Bioconductor [15]. The probes were annotated using the biomaRt package with gene symbols from the Ensembl database [16]. After the probes were annotated, the limma package was used to perform background and normalization correction using the “normexp” and “quantile” methods, respectively. Control probes, probes without gene symbols and probes that were not expressed in at least four arrays were excluded from the dataset. The expression values from replicated probes and gene symbols were averaged using the “avereps” function. To address potential batch effects during microarray processing, we included batch as a covariate in the linear model design matrix. Cleaned data were then used for the differential expression analysis using the linear model “fit” function with a design matrix based on the group and batch variables. The resulting *P*-values were adjusted using the Benjamini and Hochberg method for controlling the false discovery rate [17]. Upon manual inspection of the data, we observed minor gene expression changes. Therefore, we selected differentially expressed genes (DEGs) using an unadjusted *P*-value cutoff of ≤ 0.001.

### Co-expression network analysis (CEMiTool)

To identify groups (i.e. modules) of co-expressed genes in our dataset, the CEMiTool R package was employed, allowing the automated identification of co-expressed genes using an unsupervised gene filtering method coupled with the automated selection of parameters, enrichment, interactome and functional analyses [18]. The data used in this analysis were normalized as described above. The CEMiTool algorithm uses expression data instead of differentially expressed gene data. To account for potential batch effects influencing the gene expression values, the Combat algorithm (part of the sva R package) was used [19]. The CEMiTool package also performs over-representation analysis for pathway identification. For this, we have used the blood transcriptome module (BTM) gene set, which is more suitable for PBMC gene expression data [20]. Modules displaying an activation pattern associated with vaccination or infection were manually identified and further analyzed.

### Estimation of immune cell composition

CIBERSORTx is a method for characterizing and estimating the cell composition of complex tissues using their gene expression profile [21]. We have used CIBERSORTx via the online platform (https://cibersortx.stanford.edu/) to assess the proportion of immune cell populations in the PBMC data. For this, we utilized the PBMC tissue-specific signature matrix from ImmuCC [22], which can identify eight different cell populations typically found in PBMCs (B cells, basophils, dendritic cells, macrophages, monocytes, neutrophils, natural killer cells and T cells) and was trained using mouse data. The normalized, batch-corrected expression data were used as input, with 500 permutations. The baseline for cell populations was generated using 16 biological replicates from various timepoints from the control group (Cont).

### Cell-type specific enrichment analysis (CSEA)

To estimate the cell composition based on the genes expressed in modules M2 and M6, we utilized Web-based Cell-type Specific Enrichment Analysis of Genes (WebCSEA) accessible at (https://bioinfo.uth.edu/webcsea/). This resource encompasses tissue-cell-type expression signatures, derived from > 5.5 million cells from 111 scRNA-seq panels of human tissues and 1355 tissue cell types from 61 different general tissues across 11 human organ systems [23]. First, we identified the human orthologous of the mouse genes from modules M2 and M6 using g:Profiler (https://biit.cs.ut.ee/gprofiler/orth). Subsequently, we conducted the analysis on WebCSEA, focusing on the top 20 general cell-type dataset and then filtered for immune-related cell type. Only the cell types enriched above the Bonferroni-corrected significance cutoff of 3.69 × 10^–5^ (considering the critical *P*-value set at *α* = 0.05 and adjusting for 1355 comparisons associated with the different tissue cell types*)* were displayed [23].

### Protein-protein interaction (PPI) network construction and hub gene identification

Protein-protein interaction (PPI) networks are tools that give sense to biological machinery through visualization of the relationship between proteins [24]. For the construction of the PPI networks, the list of gene symbols composing the M2 and M6 modules (disclosed during the CEMItools analysis) were imported into the STRING online database (version 11.5; https://string-db.org/). Disconnected nodes were excluded, and the resulting network was imported into Cytoscape (version 3.9.1) [25] for visualization and further analysis. The Cytohubba plugin [26] was used to identify the hub genes based on the Maximal Clique Centrality (MCC) ranking values.

### Data availability statement

The data presented in the study are deposited in NCBI's Gene Expression Omnibus (https://www.ncbi.nlm.nih.gov/geo/) repository, series accession number GSE164094 and GSE222444.

### Statistical analysis

Data sets were tested for normality using the Shapiro-Wilk test. Differences in worm and egg burdens were analyzed with a one-way ANOVA, followed by Tukey’s multiple comparison post-hoc test. Cellular populations estimated by CIBERSORTx were plotted, and statistical analyses were conducted using the Mann-Whitney *U*-test for multiple comparisons [false discovery rate (*Q*) 5%] and the Benjamini, Krieger and Yekutieli method. For cellular population estimation using single-cell analysis with WebCSEA, only cell types enriched above the Bonferroni-corrected significance cutoff (*P* ≤ 3.69 × 10^–5^) were displayed. For identification of differentially expressed genes, unadjusted *P*-value cutoffs of ≤ 0.001 were applied. Otherwise, differences were considered statistically significant when *P* ≤ 0.05. Graphs and statistical analyses were performed using PRISM version 9.00 software (GraphPad, San Diego, CA).

## Results

### Infected group at Day 35 presented similar liver egg burden to challenge controls at Day 49

We previously reported the high efficacy of the RA vaccine and assessed the expression profile in the PBMC of vaccinated/challenged or infected mice [12]. Aiming to expand the investigation of the gene expression profile of infected animals beyond Day 17, we analyzed PBMC samples collected 35 days after a high-dose infection with 500 cercariae (Fig. 1a). The animals exhibited an average worm burden of 157 and mean liver-trapped egg burden of ~ 3700 (Fig. 1b–d). A comparative analysis with the Chc group (infected with 120 cercariae and perfused 49 days later) revealed that the worm burden of the Inf group was ~ 5.0 times higher [ANOVA, *F*(2,41) = 21.87, *P* < 0.0001] (Fig. 1b), while the liver-trapped egg burden showed no significant difference [ANOVA, *F*(3,54) = 26.1, *P* < 0.0001] (Fig. 1c).

### Infection progression induces greater gene expression changes than vaccination

After raw data processing and cleaning, differentially expressed genes (DEGs) relative to the control group were classified with a *P*-value ≤ 0.001 (Additional file 2: Table 2). The extent to which gene expression was perturbed in PBMCs following exposure to attenuated or normal cercariae is illustrated by the volcano plots (Additional file 3: Fig. 1).

Previously, we observed that animals immunized three times had the highest number of DEGs compared to the other groups [12]. Upon reanalysis, including the Inf group at Day 35, this group now presents the largest number of DEGs (1076 DEGs, 559 up- and 517 downregulated) (Additional file 3: Fig. 1). The top 15 DEGs (both up- and downregulated) for each group and timepoint are listed in Additional file 2: Table 2.

**Fig. 2:**
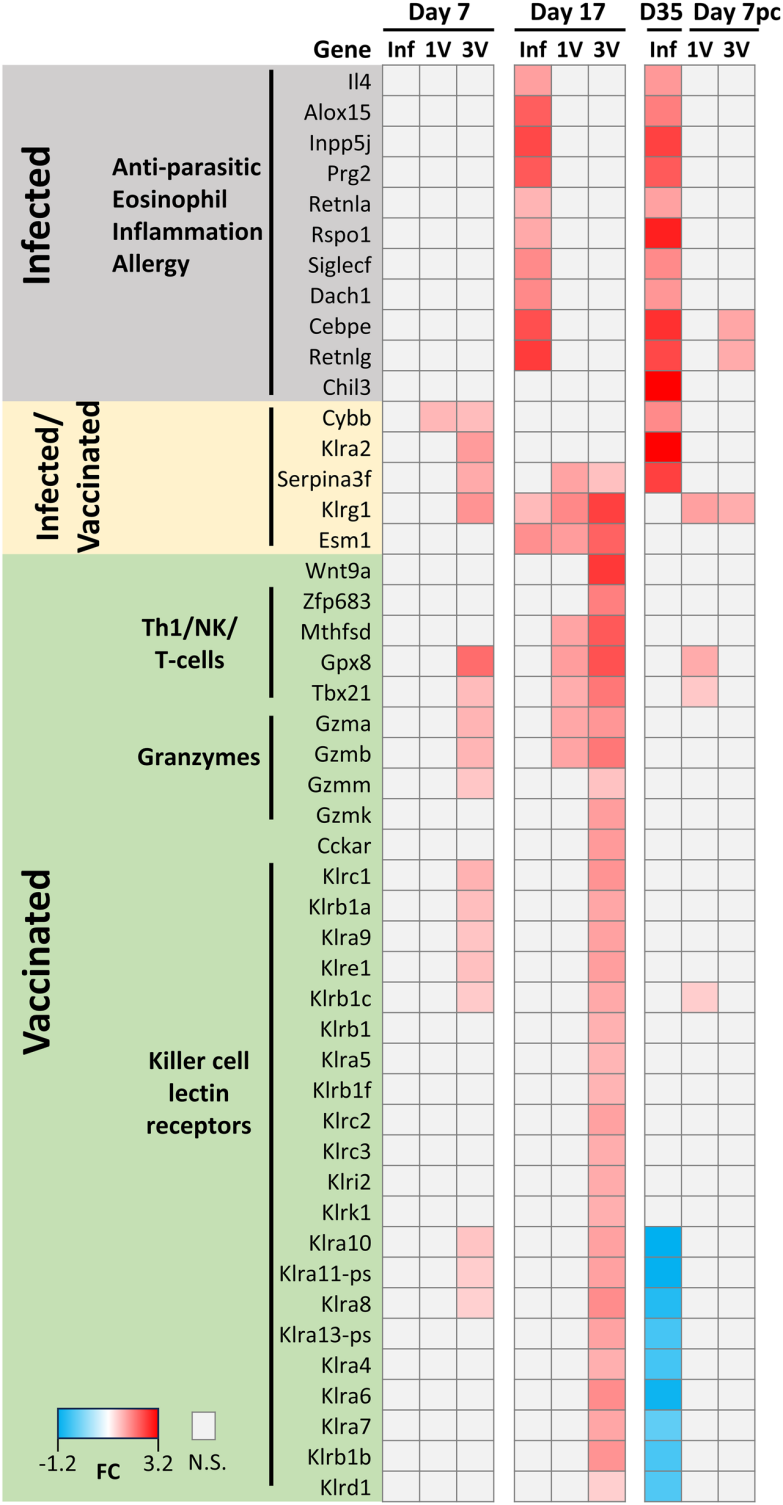
Heatmap highlighting differentially expressed genes (DEGs) in the infected and vaccinated groups as well as genes shared between both groups, which exhibit regulation at least at two timepoints. Chil 3, Wnt9a and Zfp638 were included despite being expressed at only one timepoint because they rank among the top 10 DEGs (*p* < 0.001, FDR not adjusted). Additionally, some killer lectin-like receptors (Klrs), also expressed at only one timepoint, were included because of their notable abundance

Based on these data, a heat map was generated to highlight genes associated with infection or vaccination (Fig. 2). In the Inf group, notable upregulated genes included those involved in chemotaxis in myeloid cells (Retnla and Retnlg from the Resistin family and Inpp5j), anti-parasitic defense mechanisms and eosinophil protein synthesis (Prg2; eosinophil major basic protein isoform), differentiation and functional maturation of granulocyte progenitor cells (Cebpe), eosinophil accumulation and survival (Siglecf; sialic acid binding lectin) and inflammation and allergy (Ym1/Chil3; chitinase-like 3 and Alox15).

The immunized groups, 1 V and 3 V, shared a variety of early upregulated genes associated with the activation of immune responses. These included: GPX8, a glutathione peroxidase; the Th1 transcription factor T-bet (Tbx21); various granzymes (Gmza, Gzmb, Gzmm and Gzmk); the transcription factor regulator (Zfp683); several killer cell lectin receptors (Klrs) involved in the regulation of lymphocyte and natural killer cell differentiation. Of note, some of these Klrs show negative regulation in the infected group on Day 35 (Fig. 2).

### Co-expression network analysis identified specific modules associated with infection or vaccination

**Fig. 3:**
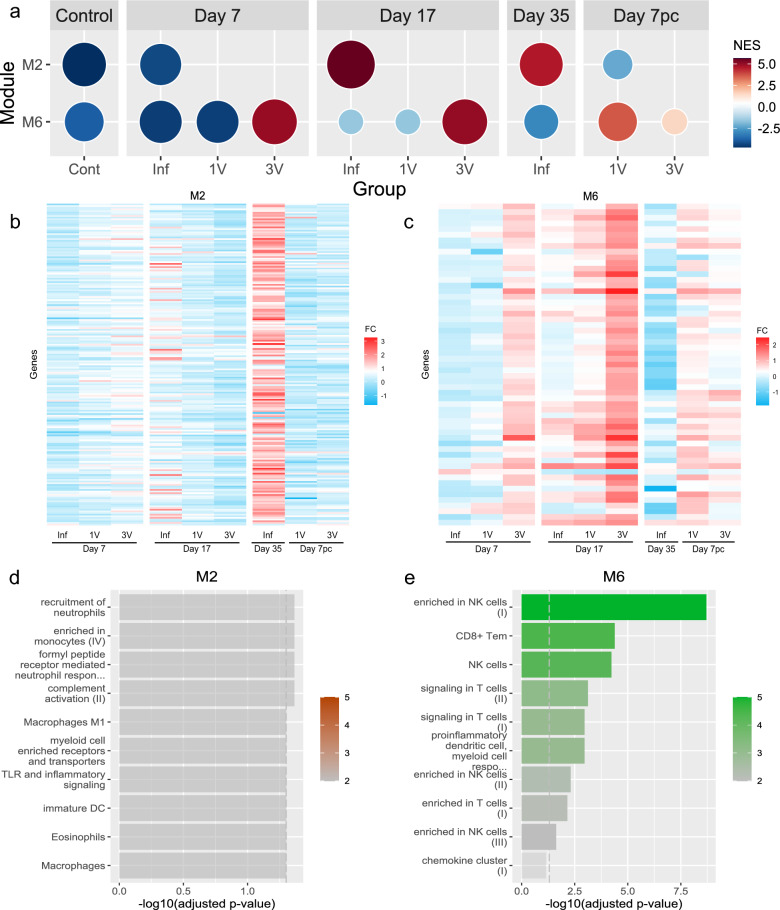
**a** Modules M2 and M6 identified by CEMiTool enrichment analysis across different timepoints, color and size denote normalized enrichment scores. **b**, **c** Heatmaps displaying the fold change of genes from modules M2 and M6, respectively. **d**, **e** Over-representation analysis based on the Blood Transcription modules (BTM), with color intensity indicating the level of significance

The CEMItool was used to identify modules of genes that are significantly co-expressed across different groups and timepoints. Among the eight identified modules, two that correlated with the infection (M2) and the vaccination (M6) processes (Fig. 3a) were pinpointed. M2, activated at Day 17 and Day 35 in the infected group, did not exhibit changes in the vaccinated groups relative to the control. On the other hand, M6 is activated in the 3 V group across all timepoints and in the 1 V group after challenge (Fig. 3a). Figure 3b and c depicts the fold change (FC) of genes that comprise both modules, showing that module activation or inhibition is coupled to the variation in gene expression. The complete analysis with expression values and the genes names are presented in Additional file 2: Table 2.

Finally, the ORA analysis using the BTM gene set uncovered a discrete enrichment of pathways related to neutrophils, macrophages and monocytes for the M2 module (Fig. 3d). In the M6 module, pathways related to enrichment of CD8 + T cells, T cell signaling and natural killer cells emerged (Fig. 3e), with the latter presenting the highest enrichment score.

### Immunization increases the proportion of natural killer cells after challenge

To estimate the composition of cell populations across the different groups, the CIBERSORTx algorithm was used with the ImmuCC peripheral blood signature matrix. To facilitate the analysis and comparisons, only the cell populations exhibiting significant differences between groups are presented (Additional file 4: Fig. 2). The Mann-Whitney *U*-test was used to identify significant differences between vaccinated and infected versus control groups. On Day 7, only the Inf group displayed significantly altered proportions, presenting increased numbers of T cells (Mann-Whitney-Wilcoxon test, adj. *P* = 0.016) compared to the control. By Day 17, 3 V showed reduced levels of B cells (Mann-Whitney-Wilcoxon test, adj. *P* = 0.006) with increased numbers of macrophages (Mann-Whitney-Wilcoxon test, adj. *P* = 0.009) and NK cells (Mann-Whitney-Wilcoxon test, adj. *P* = 0.001). Following the challenge (Day 7 pc), the immunized groups exhibited the highest NK cell levels (Mann-Whitney-Wilcoxon test, 1 V adj. *P* = 0.001 and 3 V adj. *P* = 0.002). In contrast, the Inf group exhibited an abundance of macrophages only (Mann-Whitney-Wilcoxon test, adj. *P* = 0.007).

### Cell profile using single cell databases

Additionally, we estimated the cell profiles of the M2 (infection) and M6 (vaccination) modules using WebCSEA and single-cell databases (Additional file 5: Fig. 3). M2 exhibited a higher presence of innate response-related cells, such as macrophages, monocytes and dendritic cells (Additional file 5: Fig. 3a), whereas M6 displayed an increased number of natural killer cells, corroborating the CibersortX data (Additional file 5: Fig. 3b).

### PPI network and hub gene identification

**Fig. 4:**
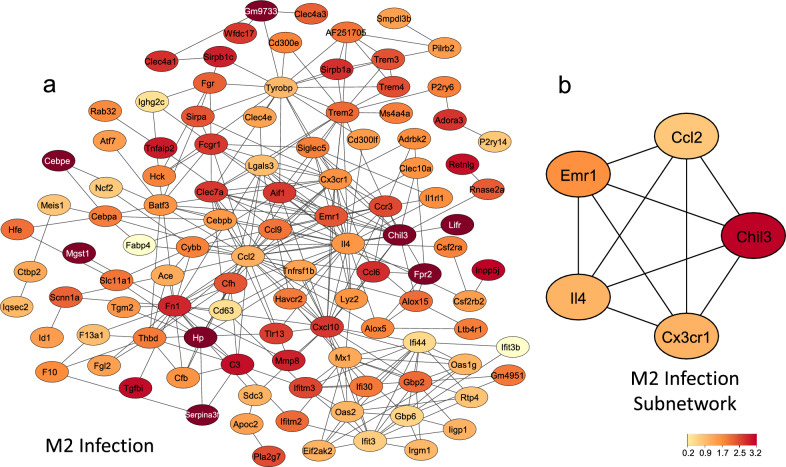
**a** Protein-protein interaction network constructed using the genes from M2 in the STRING online database. **b** Subnetwork of the five most connected genes identified by CytoHubba using the MCC algorithm. Color intensity represents the fold change of these genes in infected mice 35 days post-infection

**Fig. 5:**
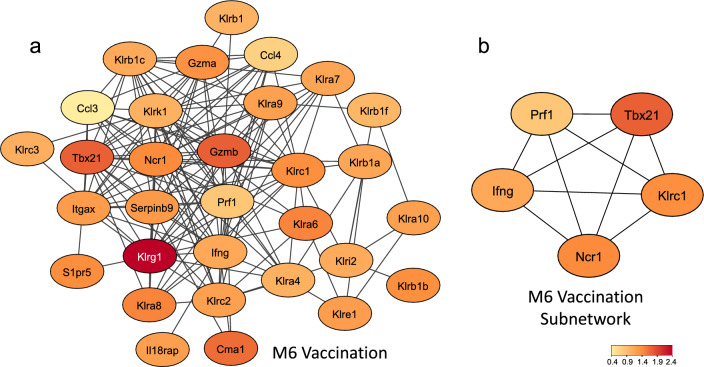
**a** Protein-protein interaction network constructed using the genes from M6 module in the STRING online database. **b** Subnetwork of the five most connected genes identified by CytoHubba using the MCC algorithm. Color intensity represents the fold change of these genes in mice 17 days after receiving three vaccinations

PPI networks for the M2 and M6 modules were constructed using the STRING online database and visualized with Cytoscape. The M2 network (Fig. 4a) included 107 nodes and 307 edges, and the M6 network (Fig. 5a) included 31 nodes and 155 edges. The top five hub genes according to the Cytohubba MCC ranking were identified for the M2, namely Chil3, Il4, Cx3cr1, Emr1 and Ccl2 (Fig. 4b), and for M6, namely Prf1, Klrc1, IFN-γ, Ncr1 and Tbx21 (Fig. 5b) networks.

## Discussion

Schistosomiasis remains a significant problem in developing countries, affecting around 200 million people worldwide [1]. While treatment options exist and are effective, helping decrease the prevalence over the last decade [2], the disease continues to be a serious public health problem. The development of an effective vaccine would be the ideal tool for prevention and eradication. However, despite many efforts, the results so far have been disappointing. The irradiated cercariae vaccine model is widely recognized as the benchmark for investigating the immune mechanisms underlying robust protection [7].

It is well established that a single exposure to the RA vaccine in mice triggers a robust Th1 response that impedes the migration of challenged schistosomula through lung capillary beds. Notably, this response does not involve cytotoxic killing of larvae, as reviewed in [3]. Subsequent vaccine exposures amplify the humoral component of protection. Elevated antibody levels produced through these additional exposures can confer passive protection on naïve recipients, as demonstrated by [27]. In both cases, IFN-γ and TNF-α are key molecules associated with protection. The effector cells are largely CD4 T-cells and macrophages that contribute to the effector foci development in the lungs of vaccinated mice around migrating challenge parasites (reviewed in [28]). While key antigens have been identified [27], replicating similar immune responses with current vaccine platforms remains a challenge despite extensive research.

To illuminate this question, we have previously generated transcriptomic data of PBMC from vaccinated C57BL/6 mice with one or three doses of irradiated cercariae as well as infected mice with 500 normal cercariae. Our previous study confirmed that there was a greater extent of leukocyte recruitment to the lungs of both the 1 V and 3 V groups compared to the infected mice. Indeed, the Inf and Chc groups exhibited a lower degree of gene perturbation at all timepoints, suggesting a weaker secondary response elicited by normal parasites [12].

Herein, we expand our analysis of the infection process by including PBMC samples collected 35 days after infection. There was a concern that a heavy infection with 500 cercariae might lead to a substantial egg burden, potentially overwhelming the mice's immune systems. To address this, we limited the analysis of the infection process to Day 35. The results showed a comparable number of eggs trapped in the liver of the Inf group compared to the moderate infection Chc group. Therefore, the substantial number of DEGs observed in the Inf group at Day 35 was not attributed to an overwhelming effect of the egg load.

Our previous analysis primarily emphasized gene set enrichment analysis using broad gene sets such as Hallmark, Reactome and Kegg [12]. In contrast, this study adopted a different approach, focusing on co-expression modules, over-representation analysis using Blood Transcription Module (BTM) gene sets and network gene hubs.

CEMiTools analysis identified modules M2 and M6, associated with infection and vaccination, respectively. ORA analysis using the BTM gene set revealed enriched pathways for both modules. M2 transcripts were linked to pathways involving neutrophils, macrophages, dendritic cells and eosinophils. However, the *P*-values associated with these data were close to the limit of significance, raising questions about the specificity of these pathways. We attempted to further explore the biological function of M2 with ingenuity pathway analysis (IPA), but the outcomes were equally generic and lacked significance (data not shown).

M2 consists of 195 genes, and it is puzzling that this relatively large gene set has not yielded a clear biological function. We hypothesize that this might be due to a lack of *Schistosoma*-specific gene sets in current datasets, potentially explaining why the algorithms failed to identify significant pathways related to the M2 molecular signature.

Recognizing the significance of protein-protein interactions (PPI) in biological systems, we constructed a PPI network to visualize the modules’ interactome and identify essential hub proteins based on their node degree [29]. The M2 hub is mainly associated with a Th2 immune response as evidenced by the presence of IL-4, a major player in Th2 polarization and M2 macrophage differentiation, which can induce the expression of other genes and markers [30]. This effect is reflected by the presence of genes such as the chemokine Ccl2, chemokine receptor Cx3cr1, lectin Chil3 and macrophage surface marker Emr1 (F4/80). These genes were described by Nascimento et al. [31] in a study on monocyte recruitment and M2 macrophage polarization in granulomatous liver tissue from mice infected with *S. mansoni*. Immune cell composition analysis conducted using CIBERSORTx predicted increasing numbers of macrophages from Day 7 to Day 35 post-infection in the blood. However, it remains to be investigated whether these macrophages exhibit the same phenotype as those described by Nascimento.

In a recent study, Houlder et al. (2023) [32] observed an increase in Remla, IL-4 and Chil3 (Ym-1) cytokines in bronchoalveolar lavage (BAL) samples collected 21 days after infecting mice with 180 cercariae. Similarly, we observed the upregulation of these genes (clustered in the M2 module) in PBMC at Day 17, where IL-4 and Chil3 appeared as hub genes. These findings indicate that the Th2 inflammatory response induced by larval migration, observed in the lungs by Day 21 [32], aligns with a systemic response detectable in PBMC, as observed here and already noted by Day 17, preceding oviposition. This implies that components from juvenile worms (17 days old) could drive this Th2 response. Although egg production is the main driver of type 2 immune responses in schistosomiasis [33], some studies have shown that antigen-specific type 2 responses, characterized by IL-4 production, can already be detected in prepatent infections (4 weeks) or in single-sex *S. mansoni* infections. This indicates that the type 2 response can arise even without exposure to eggs [32, 34, 35].

The M6 module exhibited distinct characteristics, featuring enriched pathways related to NK cells, CD8^+^ T cells and T cell signaling, all with notably significant enrichment scores. Hub genes within the M6 vaccination module are associated with the induction of cytotoxicity (Prf1, Ncr1 and Klrc1), Th1 cytokine (IFN-γ) and Th1 transcription factor T-bet (Tbx21). The gene Tbx21 offers crucial insights into the immune events occurring in vaccinated animals. This gene serves as a key transcription factor for Th1 cells, governing their development, regulating IFN-γ production, facilitating the activation of NK cells and CD8 + T cells and inhibiting the development of Th2 and Th17 cells [36]. Since it shows differential expression exclusively in vaccinated animals, particularly prominent in the 3 V group, we can speculate that the attenuated cercariae can trigger the expression of this transcription factor. Such induction would likely play a significant role in driving the Th1 arm in the highly protected 3 V group, thereby stimulating the maturation of NK and CD8^+^ T cells, consequently culminating in IFN-γ production. The CIBERSORTx and WebCSEA analysis further supports an increase in the proportions of NK cells in the 3 V group on Day 17 as well as in the 1 V and 3 V groups after challenge.

The role of NK cells in the response to the irradiated vaccine has remained relatively underexplored. Mountford et al. (1998) demonstrated that immunization of mice with soluble antigens from the lung stage, along with IL-12, resulted in significant protection against challenge. This response is characterized by a polarized Th1 response, marked by abundant IFN-γ secretion and an absence of IL-4. This mechanism likely operates through IFN-γ production by NK cells.

## Conclusions

The co-expression network analysis presented here revealed a specific vaccination module associated with Th1 activation, NK cells and CD8 + T cell cytotoxicity. Our data highlight the potential involvement of NK cells in bolstering the response to the RA vaccine via IFN-γ production regulated by the T-bet transcription factor (Tbx21). These findings may provide a basis for predicting favorable outcomes in the development of a vaccine against schistosomiasis.

## Supplementary Information

Additional file 1: Table 1. Data on parasite burden and the number of PBMC cells processed individually and after pooling for RNA extraction (RNA quality assessment) and microarray assays.

Additional file 2: Table 2. Final list of genes/probes used to identify DEGs in the reanalyzed PBMC microarray dataset, including genes from the M2 and M6 modules identified by CEMiTool.

Additional file 3: Fig. 1 Volcano plots were generated using PBMC microarray data to illustrate differentially expressed genes (DEGs) [absolute Log2(FC) > 0, *P* value ≤ 0.001, FDR not adjusted] across various timepoints in the one-vaccine dose (1 V), three-vaccine doses (3 V) or Infected (Inf) group.

Additional file 4: Fig. 2. Box plots (10–90 percentile) representing the predicted proportions of immune cell across different groups at all timepoints were generated by CIBERSORTx. Significant differences between groups were identified using Mann-Whitney U-test and *P* adj ≤ 0.05 is plotted.

Additional file 5: Fig. 3. Cell type prediction using genes from modules M2 (a) and M6 (b) with WebCSEA. Each point in the Jitter plots represents a tissue cell type, with the y-axis indicating the significance of detection as – log10 (combined *p*-value). The dashed red line represents the Bonferroni-corrected significance cutoff (*P* = 3.69 × 10^–5^) across 1355 tissue cell types.

## Data Availability

All data generated or analyzed during this study are included in this published article and additional files.
