# Supplementary figures and images for: Co-expression gene module analysis in response to attenuated cercaria vaccine reveals a critical role for NK cells in protection against Schistosoma mansoni
Source: Parasit Vectors. 2024 Nov 19;17:476. doi: 10.1186/s13071-024-06505-0 (PMC11575109; doi:10.1186/s13071-024-06505-0)

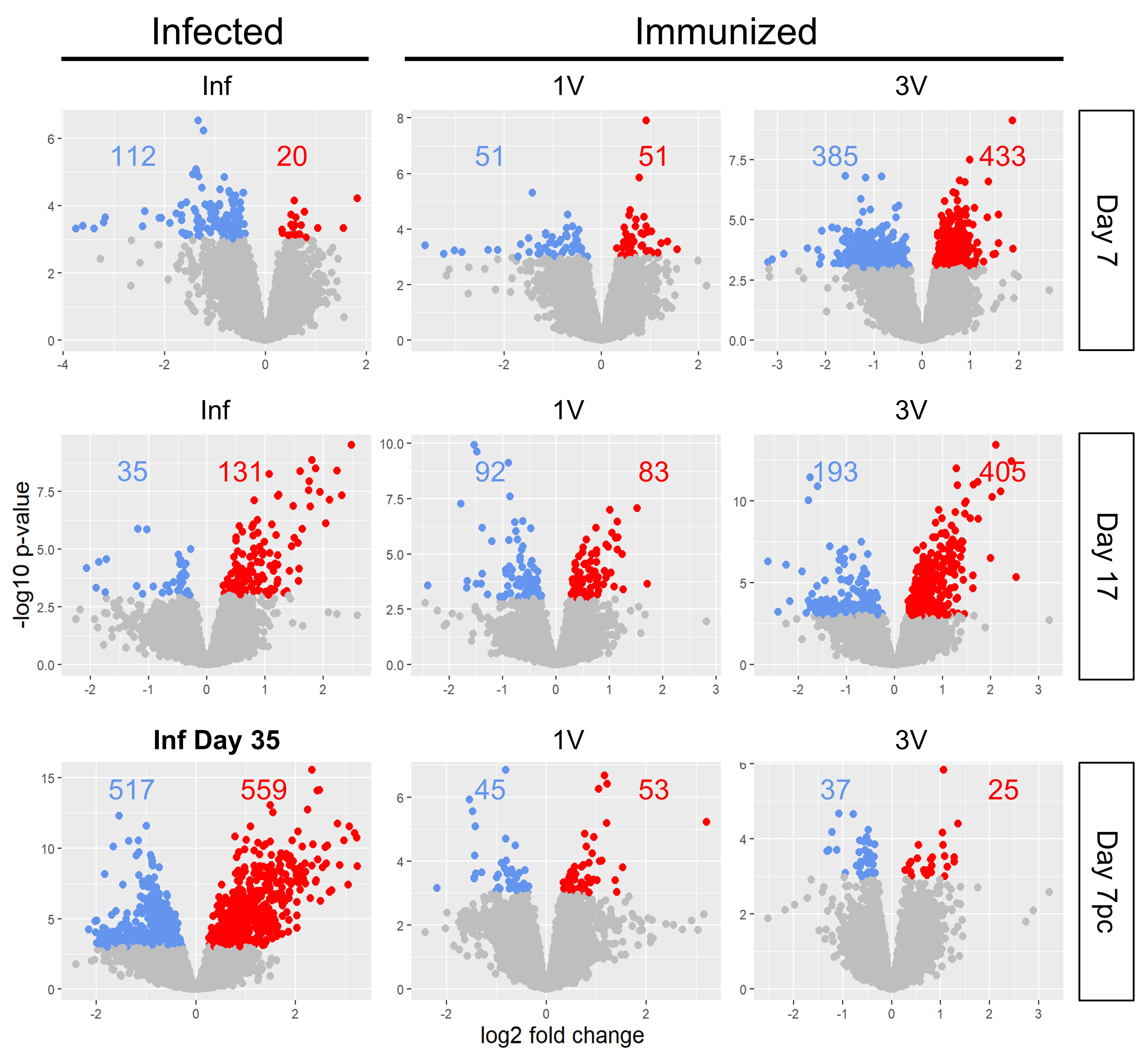

Supplement: Supplementary file 3 — Additional file 3: Fig. 1 Volcano plots were generated using PBMC microarray data to illustrate differentially expressed genes (DEGs) [absolute Log2(FC) > 0, P value ≤ 0.001, FDR not adjusted] across various timepoints in the one-vaccine dose (1 V), three-vaccine doses (3 V) or Infected (Inf) group. [file 13071_2024_6505_MOESM3_ESM.tif]

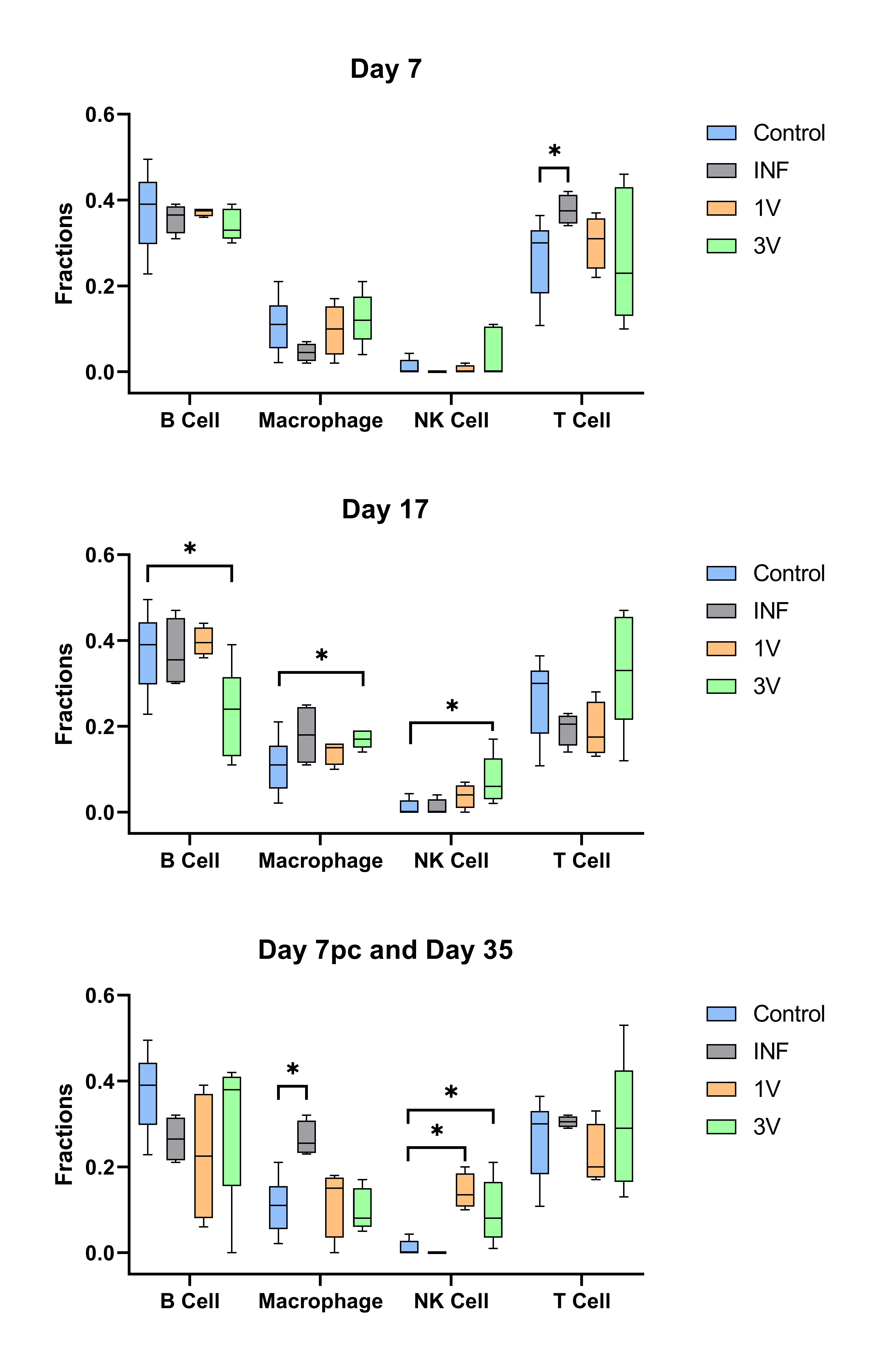

Supplement: Supplementary file 4 — Additional file 4: Fig. 2. Box plots (10–90 percentile) representing the predicted proportions of immune cell across different groups at all timepoints were generated by CIBERSORTx. Significant differences between groups were identified using Mann-Whitney U-test and P adj ≤ 0.05 is plotted. [file 13071_2024_6505_MOESM4_ESM.tif]

**a****M2**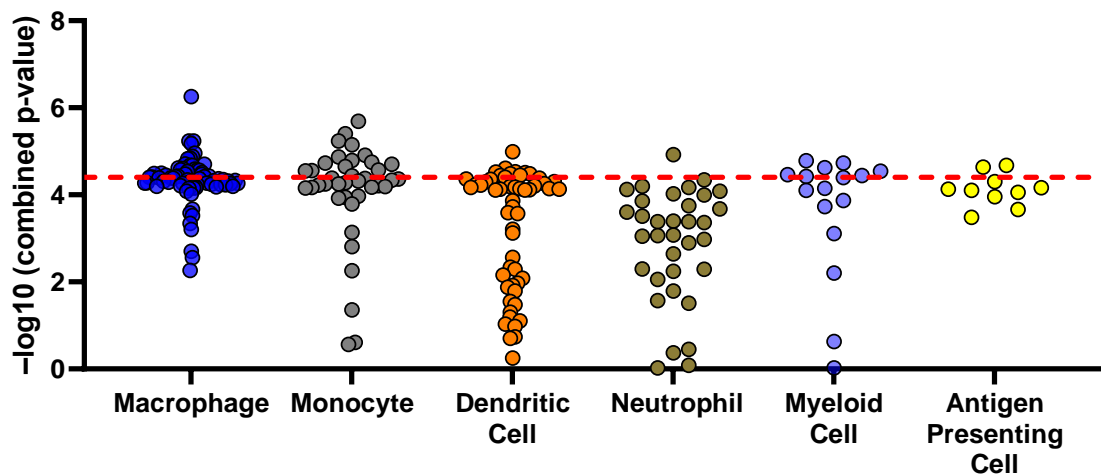**b****M6**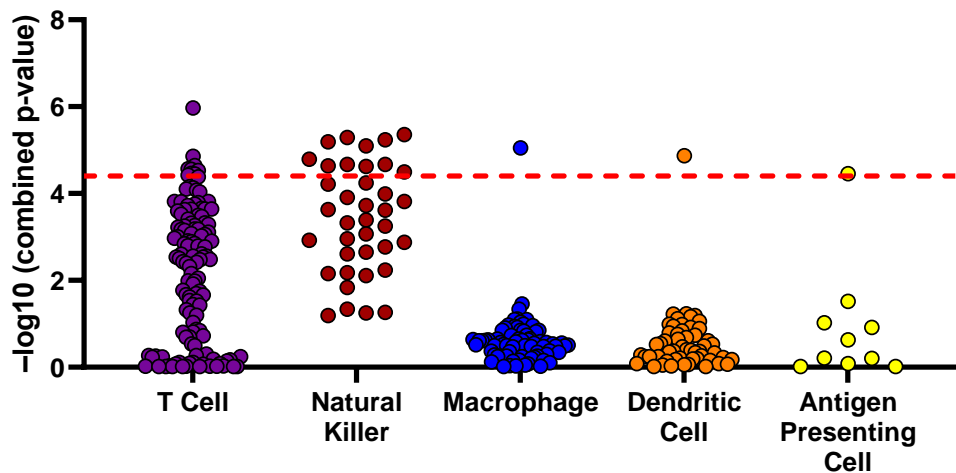

Supplement: Supplementary file 5 — Additional file 5: Fig. 3. Cell type prediction using genes from modules M2 (a) and M6 (b) with WebCSEA. Each point in the Jitter plots represents a tissue cell type, with the y-axis indicating the significance of detection as – log10 (combined p-value). The dashed red line represents the Bonferroni-corrected significance cutoff (P = 3.69 × 10^–5^) across 1355 tissue cell types. [file 13071_2024_6505_MOESM5_ESM.pdf]
